# Down regulation of *ADAM33* as a Predictive Biomarker of Aggressive Breast Cancer

**DOI:** 10.1038/srep44414

**Published:** 2017-03-15

**Authors:** Graciele C. M. Manica, Caroline F. Ribeiro, Marco A. S. de Oliveira, Isabela T. Pereira, Andressa Chequin, Edneia A. S. Ramos, Liliane M. B. Klassen, Ana Paula M. Sebastião, Larissa M. Alvarenga, Silvio M. Zanata, Lucia De Noronha, Iris Rabinovich, Fabricio F. Costa, Emanuel M. Souza, Giseli Klassen

**Affiliations:** 1 Department of Basic Pathology, Federal University of Parana, Curitiba, Paraná, Brazil; 2 Department of Biochemistry, State University of Maringa, Maringa, Brazil; 3 Department of Medical Pathology, Federal University of Parana, Curitiba, Parana, Brazil; 4 School of Medicine, Pontifical Catholic University of Parana, Curitiba, Paraná, Brazil; 5 Department of Tocogynecology, Federal University of Parana, Curitiba, Parana, Brazil; 6 Genomic Sciences and Biotechnology Program, University Catholic of Brasilia, DF, Brazil; 7 Department of Biochemistry and Molecular Biology, Federal University of Parana, Curitiba, Parana, Brazil

## Abstract

Breast cancer is a heterogeneous disease with differences in its clinical, molecular and biological features. Traditionally, immunohistochemical markers together with clinicopathologic parameters are used to classify breast cancer and to predict disease outcome. Triple-negative breast cancer (TNBC) is a particular type of breast cancer that is defined by a lack of expression of hormonal receptors and the HER2 gene. Most cases of TNBC also have a basal-like phenotype (BLBC) with expression of cytokeratin 5/6 and/or EGFR. A basal marker alone is insufficient for a better understanding of the tumor biology of TNBC. In that regard, the *ADAM33* gene is silenced by DNA hypermethylation in breast cancer, which suggests that ADAM33 might be useful as a molecular marker. In the present study, we have produced monoclonal antibodies against the ADAM33 protein and have investigated the role of ADAM33 protein in breast cancer. We used 212 breast tumor samples and lower levels of ADAM33 were correlated with TNBC and basal-like markers. A lower level of ADAM33 was also correlated with shorter overall survival and metastasis-free survival and was considered an independent prognostic factor suggesting that ADAM33 is a novel molecular biomarker of TNBC and BLBC that might be useful as a prognostic factor.

Breast cancer, which is the most common cancer among women, is a heterogeneous disease with a distinct morphology, metastatic behavior and therapeutic response^1^,^2^,^3^. Traditionally, the expression of immunohistochemical markers, including the estrogen receptor (ER), the progesterone receptor (PR) and the epidermal growth factor receptor 2 (HER2), together with clinicopathological information have been used to classify breast cancer and to predict disease outcome^4^,^5^.

Gene expression studies have revealed different intrinsic molecular subtypes of breast cancer that are biologically and clinically distinct^6^,^7^,^8^,^9^. Approximately 75% of breast cancers express typical genes of luminal epithelial cells, such as estrogen receptor (ER) and/or progesterone receptor (PR). Luminal A (LumA) breast cancers are ER+/PR+/HER2− and have a good prognosis. Luminal B (LumB) breast cancers are ER+/PR+/HER2+ and have a higher recurrence rate and a lower survival compared with the LumA subtype. HER2+ tumors occur at a 10% frequency and are characterized by high expression of the HER2 gene (ER−/PR−/HER2+), which confers aggressive biological and clinical behavior. Triple-negative breast cancer (TNBC) is a particular type of breast cancer that comprises approximately 15% of all cases and is defined by a lack of expression of the ER, PR and HER2 genes. Most cases of TNBC (80%) also share characteristics of basal-like breast cancers (BLBCs) because the expression of basal markers, such as CK5/6 or epidermal growth factor receptor (EGFR), which are identified by gene expression profiling^4^,^8^,^10^,^11^,^12^,^13^,^14^.

However, gene expression-based assays are not readily available worldwide due to their cost and technical difficulty^4^,^10^,^15^. Based on these molecular markers, breast cancer can be classified into four basic molecular subgroups using panels of immunohistochemical markers (ER, PR, HER2, EGFR and CK5/6) in a similar way to those defined by genetic profiles^4^,^10^,^11^,^15^.

ADAM33 is a member of “A Disintegrin And Metalloprotease” (ADAM) family, which are proteins that have a complex structure with pro-, catalytic (metalloprotease), disintegrin, cysteine-rich, epidermal growth factor-like, transmembrane and cytoplasmic domains^6^,^17^. One particular feature of proteins of the ADAM family is that these they show both proteolytic activity and cell adhesion properties, which means they are good candidates for the mediation of both the remodeling of the extracellular matrix (ECM) and changes in cell adhesion that characterize certain pathological processes such as tumor development^18^,^19^,^20^,^21^.

Several members of the ADAM family including ADAM9, ADAM10, ADAM12, ADAM15, ADAM17 and ADAM23 have been implicated in the pathogenesis and progression of cancer, which occurs via the cleavage of different components, the direction of cell migration and the control of various signaling pathways that are activated in cancer cells^22^,^23^,^24^,^25^,^26^,^27^.

In particular, ADAM33 has been found to be associated with asthma development and progression^28^,^29^ and to function in smooth muscle tissue remodeling^30^. In airway epithelium, it was observed that the expression of ADAM33 could be silenced by promoter hypermethylation^31^. ADAM33 plays a key role in gastric cancer pathogenesis via the up-regulation of IL-18 secretion, which results in increased cell migration and proliferation^32^. In addition, ADAM33 is involved in the *KIT* oncogene pathway in cancer, given that the ADAM33 catalytic domain is capable of cleaving stem cell factor (SCF) (Kit ligand) *in vitro*^33^. There are also indications that ADAM33 exerts a inhibitory effect on the migration of vascular smooth muscle cells in atherosclerotic lesions^34^.

In a previous study, we found that ADAM33 displayed differential expression in breast cancer tissues by methylation-specific PCR (MSP)^35^, which encouraged us to examine the importance of the expression of this protein in breast cancer. The aim of the current study was to produce a monoclonal antibody against ADAM33 to evaluate ADAM33 protein expression in breast cancer and to determine its correlation with the clinicopathological features and the prognosis of patients with breast cancer. The immunohistochemistry panel was chosen to include breast cancer-specific markers that are well-established markers of different types of breast tumors. This panel was used to compare the expression of these markers with ADAM33 expression, which was decreased in TNBC and BLBC.

## Materials and Methods

### Ethical Approval

The present study all animal experiments was approved by the Ethics Committee of the Federal University of Parana (UFPR) (Process 23075.010136/2010-20) and were performed in accordance with relevant guidelines and regulations.

The paraffin-embedded tumor tissues were obtained from breast cancer patients, the methods were carried out in accordance with the approved guidelines and all patients provided informed consent. Our study protocol was independently reviewed and approved by the institutional ethics committee of Pontifical Catholic University of Paraná (Process number 0003469-2009; CONEP Register 5365; CONEP Protocol 0480.084.000-09).

### Cell Culture

Breast cancer cell lines (PMC42, MCF7, SKBR3, MDA-MB-231 and MDA-MB-436) were cultured in RPMI 1640 medium (Gibco, USA) supplemented with 10% fetal bovine serum (FBS) (Gibco, USA), 2 mM glutamine and 40 mg/mL garamycin. PMC42 and MCF7 cells were supplemented with 0.01 mg/ml human recombinant insulin.

### Expression of the Recombinant ADAM33 Protein Coding Cysteine-Rich Domain

The amplification of *ADAM33* by RT-PCR was performed using the forward primer 5 ‘ACG GCT ACC TGG TAC CAC C and the reverse primer 5′ GCA GGA AGG CAT TGT GGT TT. The coding region of human ADAM33 was cloned into a pGEMT Easy Vector (Promega, USA). The plasmid obtained was digested with *Eco*RI (Promega, USA) and the insert was subcloned into the vector pET28a (Merck Millipore, DE). The expression of the recombinant ADAM33 protein was induced in *E. coli* BL21Ai using arabinose; in addition, a Western blot (WB) assay was used to confirm its expression with an anti-poly-histidine antibody. The protein was purified using the HisTrap Ni-Chelating column and the purified ADAM33-Rec protein was prepared as an in-gel digestion using trypsin for analysis by matrix-assisted laser desorption/ionization and time-of-flight mass spectrometry (MALDI-TOF-MS and MS/MS).

### Immunization and preparation of hybridomas

Four BALB/c female mice were immunized with the purified ADAM33-Rec protein in complete Freund’s adjuvant (each ml of contains 1 mg of heat-killed and dried *Mycobacterium tuberculosis*, 0.85 ml paraffin oil and 0.15 ml of mannide monooleate) (Sigma-Aldrich, USA). Hybridoma clones that produced antigen-specific antibodies were first screened by ELISA^36^. Cells from ELISA-positive wells were cloned at least twice by limited dilution.

The hybridoma supernatants were screened by western blotting analysis to identify the specificity of the monoclonal antibody for endogenous ADAM33 using five human breast cancer cell lines (PMC42, MCF7, SKBR3, MDA-MB-231 and MDA-MB-436). The cells were washed three times with ice-cold PBS and suspended in 200 μL lysis buffer (50 mM Tris-HCl, 120 mM NaCl, 0.5% Nonidet P-40) containing protease inhibitor (Kit Halt Thermo Scientific, USA). The lysates were separated by centrifugation at 10,000X g for 10 min at 4 °C and 100 μg of total protein was resolved by SDS-PAGE and transferred to polyvinylidene difluoride (PVDF membranes; GE Healthcare, UK). The membranes were blocked with 5% non-fat dry milk in TBS (10 mM Tris-HCl (pH 7.6), 150 mM sodium chloride) and then incubated with the primary antibody anti-ADAM33 (hybridoma supernatants and 2 ng/mL of purified mAb in TBS at a ratio of 1:2) overnight at 4 °C. The membranes were incubated for 2 hours with horseradish peroxidase-conjugated secondary anti-mouse IgG (GE Healthcare, UK) diluted 1:4,000 in TBS. The immunoreactivity was detected using the enhanced chemiluminescence (ECL) system (GE Healthcare, UK) according to the protocol recommended by the manufacturer.

### Immunocytochemistry (ICC)

Cytospin smears were prepared from three cultured breast cancer cell lines (PMC42, MCF7, SKBR3, MDA-MB-231 and MDA-MB-436) to evaluate the reactivity of the anti-ADAM33 monoclonal antibody with human ADAM33 native protein in human breast cancer cells. The prefixed unstained cytospin smear, in 95% ethanol, was incubated with the primary antibody against ADAM33 at a dilution of 0.2 μg/mL of the purified antibody, in a humidified chamber at room temperature for one hour. Incubation with the secondary antibody (Dako Advance HRP System, DakoCytomation, Inc., USA) was performed for 30 min. The incubation with 3,3′-diaminobenzidine and hydrogen peroxide substrate (DakoCytomation, Inc., USA) was performed for 3 min in order to visualize positive staining.

### Characterization of the Monoclonal Antibody anti-ADAM33

After the selection of one specific hybridoma for testing, the immunoglobulin fraction of mouse monoclonal antibodies against ADAM33 was purified by the protein A/G affinity IgG kit (GE Healthcare, USA) according to the manufacturer’s instructions. Then, the isotyping was performed using the Isostrip mouse monoclonal antibody isotyping kit (Roche, Germany) according to the manufacturer’s instructions. Additionally, ELISA and Western Blotting were used to evaluate the performance of the diluted monoclonal antibody. The ADAM33-Rec (10 μg/mL) was immobilized in 96-well plates (Immuno Nunc, Thermo Fisher Scientific, Rockford, IL) for the ELISA assay, and derail dilutions were made of the purified monoclonal antibody anti-ADAM33 (0.14 μg/mL). Western blotting analysis was performed with the ADAM33-Rec protein (10 μg/mL), which was resolved by SDS-PAGE and transferred onto PVDF membranes. The primary antibody, which was the purified monoclonal antibody anti-ADAM33, was diluted from 0.14 to 0.07 μg/mL (1:500 to 1:2000).

The total RNA from the hybridoma cells was isolated by TRIzol reagent (Thermo Fisher Scientific, USA) according to the manufacturer’s instructions. The variable heavy (VH) and variable light (VL) chains were amplified from the cDNA after synthesis using the High-Capacity cDNA Reverse Transcription Kit (Applied Biosystems, USA). The DNA fragment obtained was cloned into the pGEMT-easy vector (Promega, USA) according to the protocol of Fields *et al*.^37^. Sequencing was performed according to the BigDye sequencing protocol in an XL Genetic Analyzer (Applied Biosystems, USA).

### Immunohistochemistry (IHC)

The tissue microarray (TMA) blocks were serially sliced to generate 5-μm-thick sections. The sections were deparaffinized in xylene using two changes for 10 minutes each. Hydrate sections gradually through graded alcohols: wash in 100% ethanol three times for 1 minutes each, and 80% ethanol three times for 1 minutes each. The endogenous peroxidase activity was blocked using an Advance kit (Dako), with 5% hydrogen peroxide in methanol. The sections were then incubated with the primary antibody anti-ADAM33 at a dilution of 0.2 μg/mL overnight at 4 °C. They were then incubated with the secondary antibody (Dako Advance HRP System, DakoCytomation, Inc., USA) for 30 min, which was followed by incubation with 3,3′-diaminobenzidine and hydrogen peroxide substrate (DakoCytomation, Inc., USA) for 3 min to visualize positive staining. Finally, the sections were counter-stained in Harris hematoxylin. The staining procedures included a negative control (without primary antibody) and a positive control (normal lung tissue). The images were obtained using a motorized Axio Imager Z2 microscope (Carl Zeiss, DE), equipped with an automated scanning VSlide system (Metasystems, DE).

### ADAM33 Score classification by Immunohistochemistry

To evaluate the performance of the anti-ADAM33 antibody we tested its reactivity in human breast cancer tissues. Sections of tumors from 44 cases demonstrated to have methylated ADAM33, as defined by methylation-specific PCR (MSP) in a previous study by our group, were used^35^. For the IHC assays, two pathologists evaluated the immunostaining results (A.P.M.S. and L. D. N.). Human breast cancer tissue sections were classified based on total scores^38^. A final score ranging from 0 to 4 was assigned according to the immunohistochemical evaluation using 2 coefficients (Table 1). The first coefficient corresponded to the intensity scores, which represented the average intensity of the positive tumor cells as follows: (0) none; (1) weak; (2) strong. Then, an extension score was assigned, which represented the percentage of positively stained tumor cells with a cut-off value of 10% of cells. The extension score was assigned as follows: (0) none; (1) <10%, (2) >10%. The final score corresponds to the sum of the intensity score plus the extension score.

### Immunohistochemical evaluation of ADAM33 expression

For the immunohistochemical analysis, formalin-fixed, paraffin-embedded tumor tissues were obtained from breast cancer patients who were treated with primary surgery at the Nossa Senhora das Graças Hospital, Curitiba, PR, Brazil. A panel of immunohistochemical stains was performed on tissue microarrays (TMA) of 212 primary breast carcinomas (different patients from those included in Seniski *et al*. 2009). TMAs were constructed from formalin-fixed, paraffin-embedded tumor tissues. Protein expression in malignant breast tissues was detected using specific antibodies against ADAM33 that were produced in our laboratory. ADAM33 staining was classified in the breast cancer samples according to the total score, described above, the final score corresponds to following formula the sum of the intensity score plus the extension score, ranging from 0 to 4. Immunohistochemical evaluation was detected using specific antibodies against ER (Dako, Denmark), PR (Dako, Denmark), HER2 (Cell Marque, USA), EGFR (Dako, Denmark), CK 5/6 (Dako, Denmark), CK14 (Abcam, USA), CK17 (Novocastra - Leica Biosystems, UK), c-Kit (Dako, Denmark) and Ki-67 (Dako, Denmark) were also used according to the manufacturer’s instructions. The additionally information are in the Supplementary Table S1. In addition, positive and negative controls for each marker were routinely included during experiments. Immunohistochemical staining of the samples was evaluated and scored by two pathologists who were responsible for the clinicopathological data

### Statistical Analyses

The results from Statistical analyses were performed with the SPSS program (version 21.0, SPSS Inc., Chicago, Illinois, USA). Chi-square test was performed in both analyses, using the 44 samples to correlate *ADAM33* promoter methylation with ADAM33 protein expression (score 2, 3 and 4). And to determine the relationship between ADAM33 protein expression (score 2, 3 and 4) and the clinicopathologic features (age, tumor size, SBR, menstrual status at referral, lymph node status, RE, RP, HER2, EGFR, CK 5/6, CK 14, CK 17, c-KIT, Ki67, metastasis, death, histological type, tumor subclasses) of the breast cancer tissues were used the 212 samples. Statistical significance was assumed when *p* < 0.05. The overall survival was calculated from the time of diagnosis to the occurrence of death. Survival data were censored on June 30^th^ of 2015, the date on which the survival data were correlated with the death registry for the last time (178 months after the onset of the disease). Kaplan-Meier estimates are presented for the survival functions, and differences in survival were analyzed using the log-rank test. The clinicopathological characteristics that are used extensively to predict prognosis in clinical practice as well as ADAM33 expression were evaluated by univariate analysis and by multivariate Cox proportional hazards regression analyses to estimate hazard ratios (HRs) and 95% confidence intervals (95% CI) for overall survival and metastasis-free survival. All covariates with *p* < 0.05 were retained in the final model.

## Results

### Generation of hybridomas that secrete monoclonal antibodies against ADAM33

The RT-PCR amplification generated a DNA fragment with the coding region of human ADAM33 (nucleotides 1586 to 2198) (Supplementary Fig. S1A) that encompasses parts of the disintegrin and cysteine-rich domains. The PCR product was cloned into the pGEMT Easy Vector and the *Eco*RI fragment was used for sub-cloning into the pET28a plasmid. The expression of the ADAM33-Rec protein (27.7 kDa) was induced in *E. coli* BL21Ai (Supplementary Fig. S1B) and was confirmed by Western Blot with an anti-poly-histidine antibody (Supplementary Fig. S1C). The protein was purified using the HisTrap Ni-Chelating column (Supplementary Fig. S2A), which yielded 0.4 μg/μL of soluble protein.

Peptide mass fingerprinting (PMFs) (Supplementary Fig. S2B) was used to confirm the identity of ADAM33-Rec. MALDI-TOF mass spectra of in gel trypsin-digested ADAM33-Rec produced 19 peaks of which 6 matched (*m/z* 780.391; 1005.539; 1133.612; 1535.629; 1686.700; 2703.120) *in silico* digested peptides within 0.100 Da maximum mass deviation, resulting in 30% sequence cover. Furthermore, peak *m/z* 2703.120 was subjected to MS/MS and Mascot search of NCBIprot identified human ADAM33 (Mascot score *p* < 0.05) (Supplementary Fig. S2C). The results confirmed that the purified protein corresponded to the expected ADAM33-Rec.

The fusion experiments generated 186 hybridomas, which were screened by ELISA to evaluate the presence of specific anti-ADAM33 antibodies. From these, 141 hybridomas (76%) were positive for antibody secretion against the recombinant ADAM33.

### Characterization of the Monoclonal Antibody anti-ADAM33

**Figure 1:**
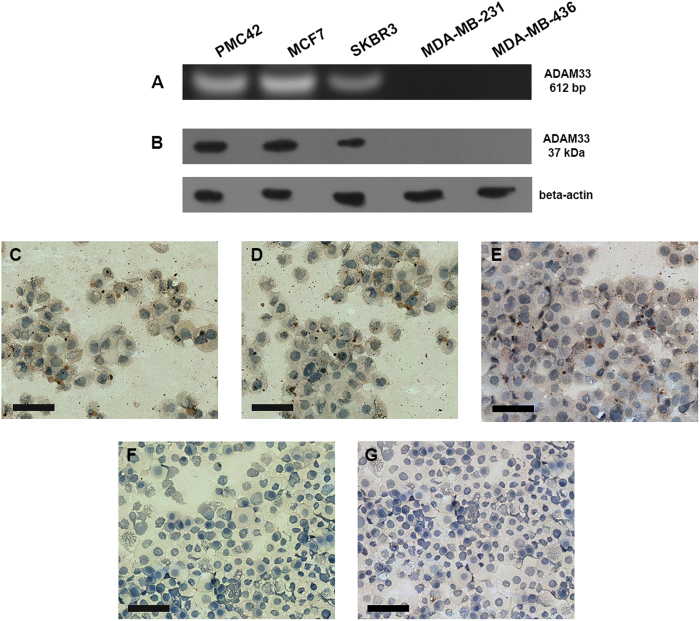
*ADAM33* expression in breast cancer cell lines. (**A**) RT-PCR of breast cancer cell lines: PMC42, MCF7 and SKBR3 cells show the amplification of a 612-bp fragment while no amplification is observed in MDA-MB-231 and MDA-MB-436 cells. (**B**) Western blotting of breast cancer cell lines using an anti-ADAM33 antibody, which shows a positive signal in PMC42 (line 1), MCF7 (line 2), SKBR3 (line 3) and no signal in MDA-MB-231 (line 4) and MDA-MB-436 (line 5). Beta-actin was used as the Western blotting control. Immunocytochemistry of breast cancer cell lines using an anti-ADAM33 antibody shows positive staining in PMC42 (**C**), MCF7 (**D**), SKBR3 (**E**) and negative staining in MDA-MB-231 (**F**) and MDA-MB-436 (**G**) cells. The IHC results are shown at X100 magnification.

Ten monoclonal antibodies (mAbs) that gave the best results in the ELISA assay were tested by Western blot and immunocytochemical assays. One of these antibodies was named GMGK06, and it was found to recognize the endogenous ADAM33 protein. We selected breast cancer cell lines that are positive (PMC42, MCF7 and SKBR3) or negative (MDA-MB-231 and MDA-MB-436) for ADAM33 protein based on *ADAM33* gene amplification by RT-PCR (Fig. 1A). The Western blot of GMGK06 revealed strong immunoreactive bands for PMC42, MCF7 and SKBR3 cells but no signal was detected in MDA-MB-231 and MDA-MB-436 cells (Fig. 1B). Furthermore, it is important to emphasize that MDA-MB-231 and MDA-MB-436 cells express other ADAM protein family members such as ADAM 9 and ADAM12^39^,^40^, which reveals the high specificity of the antibody GMGK06 to the ADAM33 protein.

Furthermore, the immunoreactivity of this monoclonal antibody was evaluated by immunocytochemistry in breast cancer cell lines. Positivity was observed in the cytoplasm of PMC42 (Fig. 1C), MCF7 (Fig. 1D) both ER positive and SKBR3 HER2 positive (Fig. 1E) breast cancer cell lines, while MDA-MB-231 (Fig. 1F) and MDA-MB-436 (Fig. 1G), triple negative, breast cancer cell lines showed no signal. GMGK06 reactivity was confirmed in human lung tissue (positive control) (Supplementary Fig. S3) because ADAM33 is strongly expressed in this tissue type^41^,^42^.

**Figure 2:**
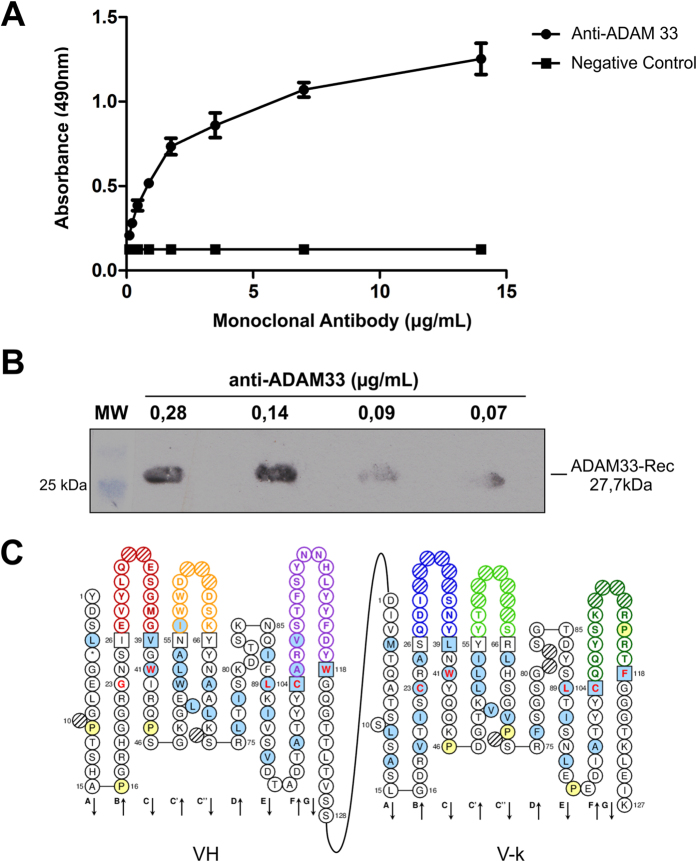
Characterization of the Monoclonal Antibody anti-ADAM33. (**A**) Dilution of the monoclonal antibody and its performance in ELISA: ADAM33-Rec (10 μg/mL) was immobilized onto plates, and the purified monoclonal antibody anti-ADAM33 was diluted from 0.14 μg/mL. (**B**) Diluted monoclonal antibody performance by Western blotting analysis with ADAM33-Rec (10 μg/mL), the purified monoclonal antibody anti-ADAM33, which was diluted from 0.14 μg/mL. (**C**) Identification of CDRs responsible for antigen-binding specificity.

Additionally, the recombinant reactivity of GMGK06 in ELISA and WB assays was determined to be 0.07 μg/mL and 0.21 μg/mL, respectively (Fig. 2A and B). Isotyping revealed that GMGK06 contains kappa light chains (V-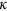) combined with an IgG1 heavy chain. According to the International Immunogenetics Information System (IMGT) database, the sequences of the functional variable heavy (VH) and variable light (VL) genes identified the framework regions (FR) and hypervariable loops (or CDRs) H1, H2, H3, L1 L2, and L3, which are responsible for interaction with the target antigen (Fig. 2C). A FASTA analysis of the protein sequence revealed strong homologies of VH and Vκ of the *Mus musculus* IgG1 antibody.

### ADAM33 Scoring System

**Figure 3:**
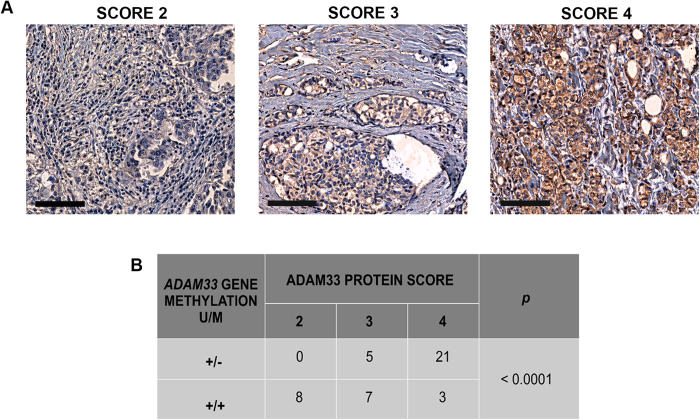
ADAM33 expression in paraffin-embedded breast cancer tissue samples. (**A**) ADAM33 scores: score of 2, low expression of ADAM33 protein; score of 3, intermediate expression of ADAM33 protein; score of 4, high expression of ADAM33 protein. The IHC results are shown at X100 magnification. (**B**) Correlation between *ADAM33* gene methylation and the ADAM33 protein score. Unmethylated promoter gene (U); Methylated promoter gene (M). Chi-square test was performed, statistical significance was assumed when *p* < 0.05.

In order to establish a scoring system for the immunoreactivity of ADAM33, an IHC assay with 44 paraffin-embedded breast cancer samples was conducted. These samples were the same as those used by our group by Seniski *et al*. (2009). Using this approach, we find three different scores for ADAM33 in our breast cancer samples: 2 (weak), 3 (intermediate) and 4 (strong), according to the staining intensity (Fig. 3A). We observe the absence of ADAM33 in breast cancer cell lines, and then we consider the possibility that had a negative tumor (score 0). However, in our cohort we do not find a sample without ADAM33 expression. This is the first study using ADAM33 as molecular marker in the breast cancer samples, we believe that more studies are needed to be assessed with a large number of samples, to evaluate if have a tumor ADAM33 score 0.

With the knowledge gained from our previous study^35^ on the ADAM33 methylation profile in the same samples (methylated or unmethylated gene promoter), we correlated the methylation status with protein expression using Chi-square test. Out of the 44 samples, 21 samples demonstrated high positivity for ADAM33 protein and had scores of 4; these samples also had the same methylation profile (unmethylated) (Fig. 3B). On the contrary, 15 samples demonstrated low ADAM33 protein, and these received scores of 2 or 3 according to our system (low ADAM33 expression); the ADAM33 gene promoter was methylated in these samples (*p* < 0.0001)^35^. In summary, we observed that samples with ADAM33 gene promoter methylation had low ADAM33 protein expression, which indicates an inverse correlation between protein expression and gene promoter methylation, as expected.

### ADAM33 expression and clinicopathological characteristics of breast cancer patients

After confirmation of the specificity and positivity of the selected monoclonal antibody (mAb) for ADAM33 using our first cohort of 44 breast cancer patients, we decided to evaluate the protein expression of ADAM33 in a different cohort of patients. The analysis of the ADAM33 protein profile in breast cancer was performed in 212 new samples that were part of a tissue microarray (TMA). Chi-square test was performed to evaluate the correlation between ADAM33 protein score and the clinicopathological data (age, tumor size, SBR, menstrual status at referral, lymph node node status, RE, RP, HER2, EGFR, CK5/6, CK14, CK17, c-KIT, Ki67, metastasis, death, histological type, tumor subclasses).

The median age of the 212 patients was 57 ± 13.83 years (range, 27 to 88 years). The histological types were either infiltrative ductal carcinoma (IDC) (n = 193; 91%) or others (infiltrative lobular carcinoma, micro papillary and tubular) (n = 19; 9%). The lymph node status of the patients was positive in 99 patients (46.7%) and negative in 113 patients (53.3%). Other clinicopathological data (e.g., menstrual status at referral, tumor size, metastasis and death) are summarized in Table 2.

**Figure 4:**
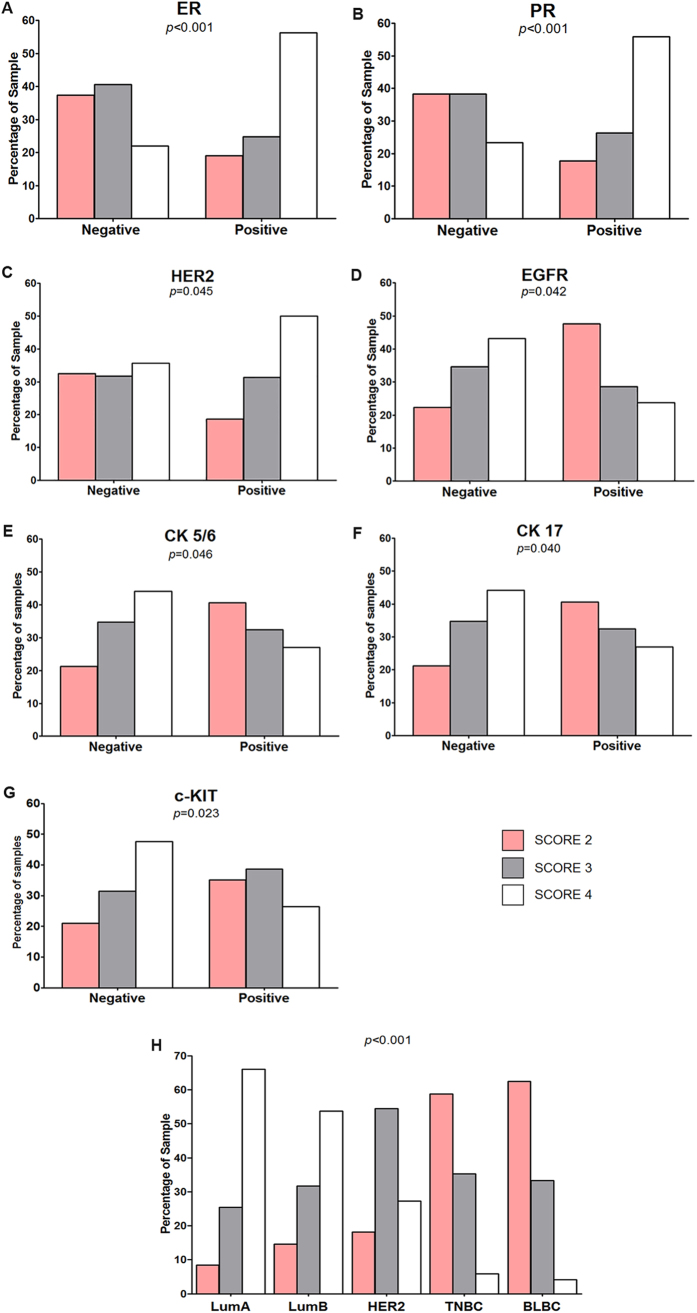
Correlation between the ADAM33 Score and biomolecular markers. Percentage of patient samples that showed a correlation between the ADAM33 score and (**A**) a ER (*p* < 0.001); (**B**) PR (*p* < 0.001); (**C**) HER2 (*p* = 0.045); (**D**) EGFR (*p* = 0.042); (**E**) CK 5/6 (*p* = 0.046); (**F**) CK17 (*p* = 0.040); (**G**) c-KIT (*p* = 0.023) and (**H**) tumor subclasses (*p* < 0.001).

The expression of ADAM33 is assigned one of three scores as described in the previous section: 2 (weak), 3 (intermediate) or 4 (strong). The ADAM33 score was not significantly associated with age (*p* = 0.679), tumor size (*p* = 0.510), histological grade according to the Scarff-Bloom-Richardson (SBR) grading scale (*p* = 0.161), menstrual status at referral (*p* = 0.498), lymph node status (*p* = 0.173) and CK14 expression (*p* = 0.416). A statistically significant difference was observed between ADAM33 expression and metastasis (*p* = 0.049), death (*p* = 0.024), histological type (*p* = 0.034) and tumor subclasses (*p* < 0.001). Moreover, the difference between the ADAM33 score and the expression of the markers of the immunohistochemical panel was significant: ER (*p* < 0.001), PR (*p* < 0.001), HER2 (*p* = 0.045), EGFR (*p* = 0.042), CK5/6 (*p* = 0.046), CK17 (*p* = 0.040), c-Kit (*p* = 0.023) and Ki67 (*p* = 0.032) (Table 2). The correlation between ADAM33 protein expression with that of other protein markers is better observed in Fig. 4. When tumors were ER+, approximately 56% of these tumors had high expression of ADAM33 (score of 4), while 78% of the samples that were ER− showed a low expression of ADAM33 (score of 2 or 3) (*p* < 0.001). A similar correlation was observed with PR, where approximately 55% of PR+ tumors expressed ADAM33 (score of 4) and 76% of PR− tumors were assigned ADAM33 scores of 2 or 3 (*p* < 0.001). HER2 expression was also negatively correlated with high ADAM33 expression, as approximately 50% of HER2+ tumors had an ADAM33 score of 4 (*p* = 0.045). When the expression of the basal-like markers EGFR, CK5/6 and CK17 was examined, we observed that tumors with the EGFR+/CK5/6+/CK17+ phenotype primarily had ADAM33 scores of 2 (47%, 40% and 36%, respectively), while tumors with the EGFR−/CK5/6−/CK17− phenotype primarily had ADAM33 scores of 4 (43%, 44% and 44%, respectively). ADAM33 expression was high in c-Kit-negative samples (47%), and 73% of these samples had ADAM33 scores of 2 or 3 compared with c-Kit-positive samples (*p* = 0.023).

Based on the biomarkers^4^,^8^,^10^,^11^ ER, PR, HER2, EGFR, CK5/6, used in this study, we stratified the samples as follows: LumA (ER+/PR+/HER2−); LumB (ER+/PR+/HER2+); HER (ER−/PR−/HER2+), BLBC (ER−/PR−/HER2−/EGFR+/CK5/6+) and the TNBC (ER−/PR−/HER2−). The ADAM33 protein correlation scores may be observed in Fig. 4H. The breast cancer samples that were ER+/PR+ had ADAM33 scores of 4, while the BLBC and TNBC subclasses showed low expression of ADAM33 (score of 2) (*p* < 0.001). These results together with the clinicopathological parameters suggested that ADAM33 might be important as a prognostic marker for patients with breast cancer.

**Figure 5:**
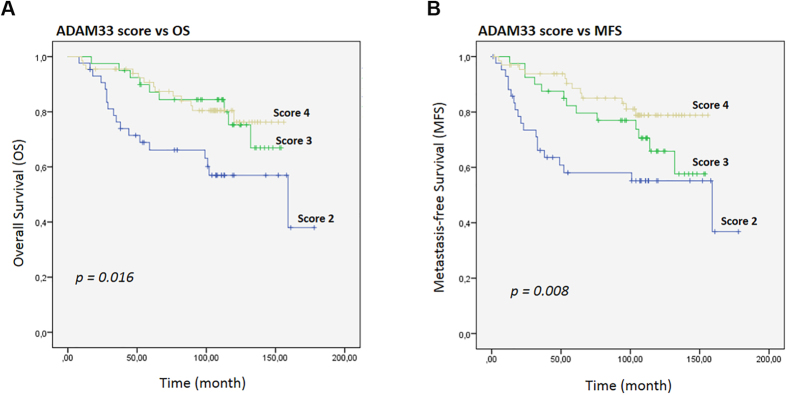
Kaplan-Meier curves for the time to breast cancer progression according to the ADAM33 Score. (**A**) Kaplan-Meier estimates are shown for overall survival and (**B**) metastasis-free survival using the ADAM33 scores. Symbols on the graph lines represent censored data; *p* values are given for log-rank tests.

In order to test this hypothesis, we evaluated the prognostic value of all the clinicopathological data of the 212 patients in a univariate analysis for overall survival (OS) and metastasis-free survival (MFS) using a Kaplan-Meier analysis (*p* value for log-rank test). The results for OS and MFS relative to the ADAM33 score are shown in Fig. 5. Both OS and MFS correlated with ADAM33 presence and absence, respectively (*p* = 0.016 and *p* = 0.008, respectively) (Fig. 5A and B). The Kaplan-Meier analysis showed that the overall survival (*p* = 0.004) and metastasis-free survival (*p* = 0.004) were significantly shorter when ADAM33 expression was lower (score of 2) when compare with other score (score 3 and 4 together) (Supplementary Fig. S5).

The next step was to analyze the effects of co-variables on OS and MFS in the Cox proportional hazard regression model. The results of the univariate analysis are shown in Table 3. In the multivariate analysis, all variables with *p* < 0.05 from the univariate analysis were selected to build a multiple model (Table 3). For overall survival, a high SBR score (*p* = 0.010), low ADAM33 expression (*p* = 0.013) and occurrence of metastasis (*p* < 0.001) were considered to indicate a poor prognosis. Besides, when grouping the scores 3 and 4 we have observed that statistical relevance of ADAM33 as an independent factor in the multivariate analysis is maintained (Supplementary Table 2). Moreover, ADAM33 expression, death and tumor subclasses (*p* = 0.021, *p* < 0.001, *p* = 0.020, respectively) were considered independent prognostic factors for MFS. Patients with ADAM33 scores of 2 had a higher risk of death (HR 0.464; 95% CI 0.253 to 0.848) and metastasis development (HR 0.581; 95% CI 0.365 to 0.923).

## Discussion

Members of the ADAM family of proteins are involved in fundamental processes such as cell adhesion, cell fusion, cell migration, membrane protein shedding and proteolysis. For this reason, it is not surprising that deregulated expression of ADAM family members has been reported in human tumors^43^. In agreement with this observation, the differential expression of the *ADAM33* gene has motivated our group to investigate if the ADAM33 protein may be used as a potential biomarker for breast cancer.

The standard clinical evaluation of breast tumors involves the immunoreactivity of various antibodies in paraffin-embedded tissue sections. Although commercial anti-ADAM33 antibodies exist, they are generated with synthetic peptides that showed no reactivity for ADAM33, which raises many doubts over the specificity of the antibodies used to detect ADAM33 in some studies^31^,^41^,^42^,^44^ and in our own experience. The specificity of an antibody is essential to evaluate the expression of a single protein, which makes it possible to perform IHC assays with greater specificity.

In order to produce better and more specific antibodies against ADAM33, we produced monoclonal antibodies from human recombinant ADAM33. The first step was to produce the recombinant ADAM33 protein, which was identified by MALDI/TOF-MS/MS; this ensured that the immunization would be specific to human ADAM33 (Supplementary Fig. S2). The mAb selection was based on the reactivity and specificity of the mAb to ADAM33 according to Western blotting and immunocytochemical assays using human breast cancer cell lines that are positive or negative for ADAM33. The human breast cancer cell lines PMC42, MCF7 and SKBR3, which are positive for ADAM33 expression (Fig. 1A), showed a positive cytoplasmic staining (Fig. 1C,D and E) similar to what was previously observed in lung tissues in which only 10% of the ADAM33 produced is directed to the extracellular membrane in airway epithelium^16^. On the contrary, no signal was observed (Fig. 1F and G) in invasive breast cancer cell lines (MDA-MB-231 and MDA-MB-436) that are negative for ADAM33.

The experimental analysis of the breast carcinomas and cancer cell lines revealed high levels of α5, β1 and β5 integrins^45^,^46^. Some studies investigated differences in integrin expression in the human breast cancer cell lines MDA-MB-231, MDA-MB-435 and MCF7, and all of them expressed high levels of α4β1 and α5β1 integrins^45^,^46^. The breast cancer cell line MDA-MB-231 is highly invasive, and blocking experiments with anti-α5 or anti-β1 integrin, as well as the specific knockdown α5-integrin, dramatically decreased the invasiveness of these cell lines into the ECM. Thus, α5β1 integrin might facilitate the tumorigenic process in breast carcinoma cells^45^. Huang *et al*. has shown that ADAM33 protein inhibited the α4β1- and α5β1-mediated migration of CHKO1 cells through the ECM^47^. Therefore, it is a plausible hypothesis that in the metastatic breast cancer cell line MDA-MB-231 the epigenetic silencing of *ADAM33* may be important in the invasion process. We are aware that ADAM33 is not the only protein that is responsible for the facilitation or the inhibition of metastasis. Because metastasis is a very complex process in tumor cells, several other players are also important in cancer progression. However, it is important to note that a single integrin can regulate many different aspects of tumor progression^48^.

*ADAM33* gene expression variation in breast tumor samples, including methylation as determined by methylation-specific PCR (MSP), has been described previously by our group^35^. In this study, samples with *ADAM33* gene promoter methylation exhibited low ADAM33 protein signal (scores of 2 or 3), whereas samples in which the *ADAM33* gene promoter was unmethylated showed strong ADAM33 protein signal (score of 4), as expected. Then, with these results, it is possible to suggest that the ADAM33 protein might be further investigated as a biomarker in breast cancer.

Extensive investigations continue the search for new biomarkers that may improve breast cancer prognosis and facilitate the implementation of new therapies. To date, cost and complexity issues have rendered gene expression profiling impractical as a routine diagnostic tool in hospitals. Moreover, the classification of breast cancer subgroups based on IHC markers is widely used in both clinical and research settings due to its reliability and reproducibility^15^,^49^,^50^. Here, we compare clinicopathological information and the expression of a panel of IHC markers (ER, PR, HER2, EGFR, CK 5/6, CK14, CK17, c-Kit and Ki67) with the ADAM33 protein scores to determine whether ADAM33 protein is clinically efficient as a prognostic or predictive marker for breast cancer.

In the current study, we observed that an ADAM33 score of 4 is directly correlated with the ER+/PR+ phenotype, low Ki67 expression and the absence of basal marker expression (e.g., EGFR, CK5/6 and c-Kit). Moreover, LumA and LumB breast carcinomas showed a strong signal for ADAM33. In addition, the absence of metastasis (*p* = 0.049) and death (*p* = 0.024) was correlated with the high expression of ADAM33. These results suggested that ADAM33 might be an important marker of good prognosis of disease because LumA and LumB breast carcinomas are less aggressive compared with the HER2+, TNBC and BLBC tumor subclasses^4^,^11^,^14^.

It is also important to report that low expression of ADAM33 in tumors was correlated with the ER−/PR− phenotype and with positivity for EGFR, CK5/6, CK17 and c-Kit; this is an important relationship because these are markers of and predictors in TNBC and BLBC. These data are in accordance with low expression of ADAM33 by IHC (score of 2), which was associated with the TNBC and BLBC tumor subclasses. Patients with TNBC and BLBC typically have a poorer outcome compared with patients with other breast cancer subtypes due to an inherently aggressive clinical behavior that often affects younger individuals, and due to a lack of recognized molecular targets for therapy^4^,^10^,^11^,^14^.

Further analysis revealed that patients whose tumors were assigned ADAM33 scores of 2 had a significantly poorer OS and DFS compared with patients whose tumors were assigned an ADAM33 score of 3 and 4 (*p* = 0.016 and *p* = 0.008, respectively) (Fig. 5A and B); this demonstrates a concordance with the results that showed that the absence of metastasis and death correlated with an ADAM33 score of 4. The molecular mechanisms facilitated by the absence of ADAM33, which results in the poor prognosis of these patients, remain obscure. Nevertheless, the decrease in ADAM33 protein might be an important mechanism of tumor progression because it has been shown to be correlated with a high risk of metastasis development (HR 0.581; 95% CI 0.365 to 0.923, *p* = 0.021) and overall survival (HR 0.464; 95% CI 0.253 to 0.848, *p* = 0.013) in our multivariate analysis (Table 3); moreover, was also found to be correlated with aggressive tumor, which corresponds to TNBC and BLBC.

In addition to, the absence or low expression of ADAM33 protein might contribute to an increase in aggressiveness and metastases, which shows that ADAM33 may play an important role in breast cancer biology. We showed here for the first time that ADAM33, in combination with currently available biomarkers, may be a novel molecular marker to better ascertain the prognosis of breast cancer. The importance of ADAM33 in TNBC and BLBC is clear and could improve our knowledge of the most aggressive breast cancer subtypes.

## Additional Information

**How to cite this article:** Manica, G. C. M. *et al*. Down regulation of *ADAM33* as a Predictive Biomarker of Aggressive Breast Cancer. *Sci. Rep.* **7**, 44414; doi: 10.1038/srep44414 (2017).

**Publisher's note:** Springer Nature remains neutral with regard to jurisdictional claims in published maps and institutional affiliations.

## Supplementary Material

Supplementary Information

## Figures and Tables

**Table 1:** Scoring System proposed for immunohistochemistry evaluation of Breast Cancers according to the immunoreactivity of ADAM33.

Intensity		Distribution	
Intensity pattern	Intensity score	Extension	Distribution score
None	0	None	0
Positive Weak	1	Focal	1
Positivo Strong	2	Difuse	2

**Table 2:** Correlations between ADAM33 score and clinicopathologic parameters of breast cancer samples.

Features	*n*	ADAM33 Score			*p* value*
		2	3	4	
**Age**					
45	42 (19.8)	13 (31)	14 (33.3)	15 (35.7)	0.6719
≥45	170 (80.2)	44 (25.9)	53 (31.2)	73 (42.9)	
**Tumor Size (cm)**					
≤2	98 (46.2)	23 (23.5)	34 (34.7)	41 (41.8)	0.510
>2	114 (53.8)	34 (29.8)	33 (28.9)	47 (41.2)	
**SBR**					
I	43 (20.3)	13 (30.2)	11 (25.6)	19 (44.2)	0.161
II	99 (46.7)	20 (20.4)	32 (32.6)	47 (48.0)	
III	70 (33.0)	24 (35.2)	24 (33.8)	22 (31.0)	
**Menstrual status at referral**					
Premenopausal	98 (65.3)	25 (25.5)	27 (27.6)	46 (46.9)	0.4985
Postmenopausal	52 (34.7)	18 (34.6)	13 (25.0)	21 (40.4)	
**Lymph node Status**					
Negative	113 (53.3)	35 (31)	30 (26.5)	48 (42.5)	0.173
Positive	99 (46.7)	22 (22.2)	37 (37.4)	40 (40.4)	
**RE**					
Negative	91 (42.9)	34 (37.4)	37 (40.6)	20 (22.0)	**<0.001**
Positive	121 (57.1)	23 (19.0)	30 (24.8)	68 (56.2)	
RP					
Negative	94 (44.3)	36 (38.3)	36 (38.3)	22 (23.4)	**<0.001**
Positive	118 (55.7)	21 (17.8)	31 (26.3)	66 (55.9)	
HER2					
Negative	126 (59.4)	41 (32.5)	40 (31.8)	45 (35.7)	**0.045**
Positive	86 (40.6)	16 (18.6)	27 (31.4)	43 (50.0)	
EGFR					
Negative	130 (86.1)	29 (22.3)	45 (34.6)	56 (43.1)	**0.042**
Positive	21 (13.9)	10 (47.6)	6 (28.6)	5 (23.8)	
CK 5/6					
Negative	118 (76.1)	25 (21.2)	41 (34.7)	52 (44.1)	**0**.**046**
Positive	37 (23.9)	15 (40.6)	12 (32.4)	10 (27.0)	
CK 14					
Negative	85 (57.4)	19 (22.4)	28 (32.9)	38 (44.7)	0.416
Positive	63 (42.6)	19 (30.2)	22 (34.9)	22 (34.9)	
CK 17					
Negative	104 (64.2)	20 (19.2)	38 (36.6)	46 (44.2)	**0.040**
Positive	58 (35.8)	21 (36.2)	20 (34.5)	17 (29.3)	
c-Kit					
Negative	105 (64.8)	22 (21.0)	33 (31.4)	50 (47.6)	**0.023**
Positive	57 (35.2)	20 (35.1)	22 (38.6)	15 (26.4)	
Ki67					
Low	98 (61.3)	21 (21.4)	28 (28.6)	49 (50.0)	**0.032**
High	62 (38.7)	19 (30.7)	25 (40.3)	18 (29.0)	
Metastasis					
Negative	128 (67.8)	30 (23.4)	31 (24.2)	67 (52.4)	**0.049**
Positive	61 (32.2)	23 (37.7)	17 (27.9)	21 (34.4)	
Death					
Negative	127 (71.3)	24 (19)	37 (29.1)	66 (51.9)	**0.024**
Positive	51 (28.7)	19 (37.3)	15 (29.4)	17 (33.3)	
**Histological Type**					
Ductal	193 (91)	48 (24.9)	60 (31.1)	85 (44)	**0.034**
Other*	19 (9)	9 (47.4)	7 (36.8)	3 (15.8)	
**Tumor Subclasses**					
Luminal A	47 (31.1)	4 (8.5)	12 (25.5)	31 (66)	**<0.001**
Luminal B	41 (27.1)	6 (14.6)	13 (31.7)	22 (53.7)	
Her2	22 (14.6)	4 (18.2)	12 (54.5)	6 (27.3)	
TNBC	17 (11.3)	10 (58.8)	6 (35.3)	1 (5.9)	
BLBC	24 (15.9)	15 (62.5)	8 (33.3)	1 (4.2)	

*Chi-square test was performed in order to correlate ADAM33 expression and the clinicopathologic features of the breast cancer tissues. Statistical significance was assumed when *p* < 0.05.

**Table 3:** Time to breast cancer progression in relation to clinicopathological characteristics: Cox proportional hazards model.

Analysis	Overall survival			Metastasis Free Survival		
	HR	95% CI	*p* value	HR	95% CI	*p* value
**Univariate**						
SBR	2.072	1.267 to 3.389	**0.004**			0.053
Size				1.909	1.010 to 3.608	**0.047**
ADAM33	0.618	0.423 to 0.904	**0.013**	0.576	0.400 to 0.829	**0.003**
ER	0.299	0.151 to 0.590	**0.001**	0.321	0.169 to 0.608	**0.000**
PR	0.259	0.129 to 0.521	**0.000**	0.281	0.147 to 0.540	**0.000**
Lymph Node	2.807	1.450 to 5.434	**0.002**	2.380	1.285 to 4.405	**0.006**
Metastasis	12.638	5.982 to 26.70	**0.000**			
Death				15.403	7.77 to 30.534	**0.000**
Tumor Subclasses	1.428	1.102 to 1.851	**0.007**	1.463	1.145 to 1.869	**0.002**
**Multivariate**						
SBR	3.075	1.304 to 7.248	**0.010**	0.937	0.515 to 1.701	0.830
Size	1.095	0.492 to 2.440	0.823	1.601	0.735 to 3.486	0.236
ADAM33	0.464	0.253 to 0.848	**0.013**	0.581	0.365 to 0.923	**0.021**
ER	0.405	0.091 to 1.805	0.236	0.382	0.080 to 1.827	0.228
PR	0.527	0.126 to 2.205	0.380	0.552	0.138 to 2.208	0.401
Lymph Node	1.541	0.686 to 3.460	0.295	1.226	0.589 to 2.554	0.586
Metastasis	20.862	7.877 to 55.256	**0.000**			
Death				13.969	6.513 to 29.69	**0.000**
Tumor Subclasses	0.433	0.237 to 1.791	0.072	0.512	0.291 to 0.900	**0.020**

Multivariate Cox proportional hazards regression analyses to estimate hazard ratios (HRs) and 95% confidence intervals (95% CI) for overall survival and metastasis-free survival. All covariates with *p* < 0.05 that were obtained in the univariate analysis were retained in the multivariate model.
