# Supplementary material for: Down regulation of ADAM33 as a Predictive Biomarker of Aggressive Breast Cancer
Source: Sci Rep. 2017 Mar 15;7:44414. doi: 10.1038/srep44414 (PMC5353751; doi:10.1038/srep44414)
Supplement: Supplementary Information [file srep44414-s1.pdf]

Down regulation of *ADAM33* as a Predictive Biomarker of Aggressive Breast Cancer

Graciele C. M. Manica[1], Caroline F. Ribeiro[1], Marco A. S. de Oliveira[2], Isabela T. Pereira[1], Andressa Chequin[1], Edneia A. S. Ramos[1], Liliane M. B. Klassen[1], Ana Paula M. Sebastião[3], Larissa M. Alvarenga[1], Silvio M. Zanata[1], Lucia De Noronha[4], Iris Rabinovich[5], Fabricio F. Costa [6], Emanuel M. Souza [7], Giseli Klassen*[1]

Affiliations:

[1]Department of Basic Pathology, Federal University of Parana, Curitiba, Paraná, Brazil;

[2]Department of Biochemistry, State University of Maringa, Maringa, Brazil;

[3]Department of Medical Pathology, Federal University of Parana, Curitiba, Parana, Brazil;

[4] School of Medicine, Pontifical Catholic University of Parana

[5]Department of Tocogynecology, Federal University of Parana, Curitiba, Parana, Brazil;

[6] Genomic Sciences and Biotechnology Program, University Catholic of Brasilia, DF, Brazil;

[7]Department of Biochemistry and Molecular Biology, Federal University of Parana, Curitiba, Parana, Brazil,

* Corresponding author

Supplementary information

Figure S1. Expression of Recombinant ADAM33 protein. (A) The human ADAM33 protein shows the coding region of nucleotides 1586 to 2197: ADAM33-Rec encompasses part of the disintegrin and cysteine-rich domains. (B) Gel SDS/PAGE of total protein that was extracted from *E. coli* BL21Ai cells with or without arabinose to test the induction of ADAM33-Rec protein: (MW) Molecular Weight, (1) *E. coli* BL21Ai non-induction and (2) induction of ADAM33-Rec protein (27.7 kDa) expression in *E. coli* BL21Ai. (C) Western blotting (WB) with an anti-poly-histidine antibody: (MW) Molecular Weight, (1) no-induction (2) induction of the ADAM33-Rec protein (27.7 kDa) in *E. coli* BL21Ai.

Figure S2. Purification, MALDI-TOF-MS and MS/MS analysis of ADAM33-Rec. (A) SDS-PAGE gel shows the purification of ADAM33-Rec. Soluble extract of ADAM33-Rec protein expressed in *E. coli.* BL21Ai was purified using a Hi-Trap Ni-Chelating column (GE Healthcare). The recombinant protein was eluted using an imidazole gradient (from 20 mM to 1 M). Samples were separated by SDS–PAGE and stained with Coomassie blue.

(B) Peptide mass fingerprinting (PMF) of ADAM33-Rec protein. The purified protein band was in-gel digested with trypsin and analyzed by MALDI-TOF-MS/MS. Peaks were considered a match when the maximum mass deviation from calculated *m/z* was 0.100 Da. Sequence cover was calculated as the ratio [number of amino acids in identified peptide]/[number amino acids of ADAM33-Rec]. Identified peaks are shown in panel B together with the matching peptides.

(C) Peak *m/z* 27032.120 was subjected to MS/MS and fragmentation mass profile used for Mascot search of NCBIprot leading to the identification of human ADAM33 ($p < 0.05$).

Figure S3. ADAM33 protein expression in paraffin-embedded human lung tissue by IHC. The IHC results are shown at X100 magnification. The primary antibody used was GMGK06, which shows a positive signal in human lung tissue (black arrows).

Figure S4. Negative control of paraffin-embedded breast cancer tissue by IHC. The IHC results are shown at X100 magnification. Breast cancer sample incubated without primary antibody.

Figure S5. Kaplan-Meier curves for the time to breast cancer progression according to the ADAM33 Score. (A) Kaplan-Meier estimates are shown for overall survival and (B) metastasis-free survival using the ADAM33 scores. Symbols on the graph lines represent censored data; *p* values are given for log-rank tests.

Table S1. Panel of Antibody specification used in Immunohistochemical evaluation.

**Antibody**		**Dilution**
Anti- ER, Monoclonal antibody, clone EP1	Dako, Denmark	Ready to use
Anti-PR, Monoclonal antibody, clone PgR 636	Dako, Denmark	Ready to use
Anti-Her, Monoclonal antibody, clone SP3	Cell Marque	Ready to use
Anti- CK 17 , Monoclonal antibody, clone E3	Novocastra - Leica Biosystems, UK	1:20
Anti-c-*Ki,* Policlonal Anti-Rabbit Code: A4502	Dako, Denmark	1:100
Anti-CK 5/6, Monoclonal antibody, clone D5/16 B4	Dako, Denmark	1:50
Anti-CK 14, Monoclonal antibody, clone LL002	Abcam, USA	1:100
Anti-EGFR, Monoclonal antibody, clone H11	Dako, Denmark	1:100
Anti-Ki67, Monoclonal antibody, clone MIB-1	Dako, Denmark	Ready to use

Table S2. Time to breast cancer progression in relation to clinic pathological characteristics: Cox proportional hazards model.

Analysis	Overall survival			Metastasis Free Survival		
	HR	95 % CI	*p* value	HR	95 % CI	*p* value
**Univariate**						
ADAM33<br>Score 2 vs (3 and 4)	0.407	0.216 to 0.769	**0.006**	0.423	0.231 to 0.777	**0.006**
**Mulivariate**						
ADAM33<br>Score 2 vs (3 and 4)	0.480	0.230 to 0.999	**0.05**	0.413	0.191 to 0.895	**0.025**

Multivariate Cox proportional hazards regression analyses to estimate hazard ratios (HRs) and 95% confidence intervals (95% CI) for overall survival and metastasis-free survival. All covariates with $p < 0.05$ were retained in the final model.

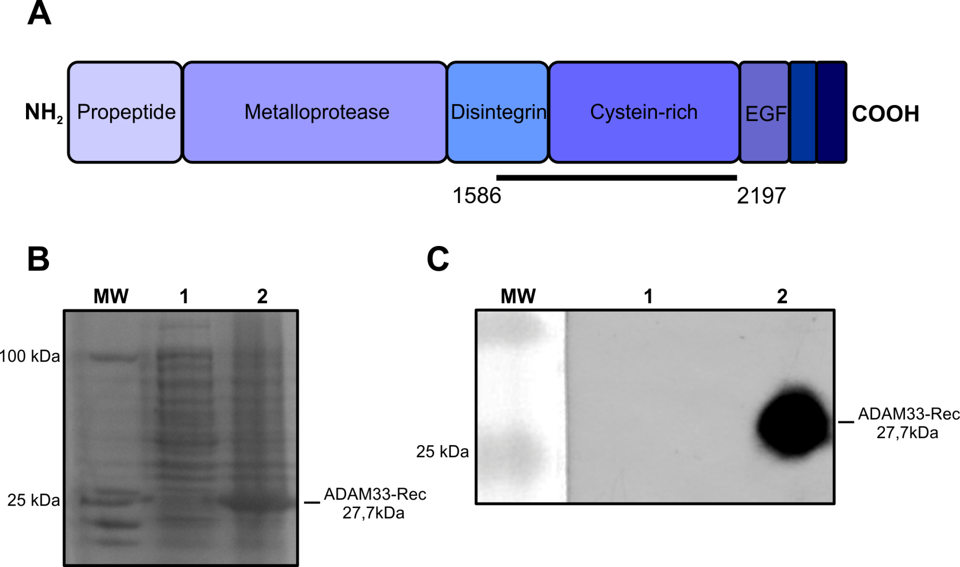
A
NH2
Propeptide
Metalloprotease
Disintegrin
Cystein-rich
EGF
COOH
1586
2197
B
MW
1
2
100 kDa
25 kDa
ADAM33-Rec
27,7kDa
C
MW
1
2
25 kDa
ADAM33-Rec
27,7kDa

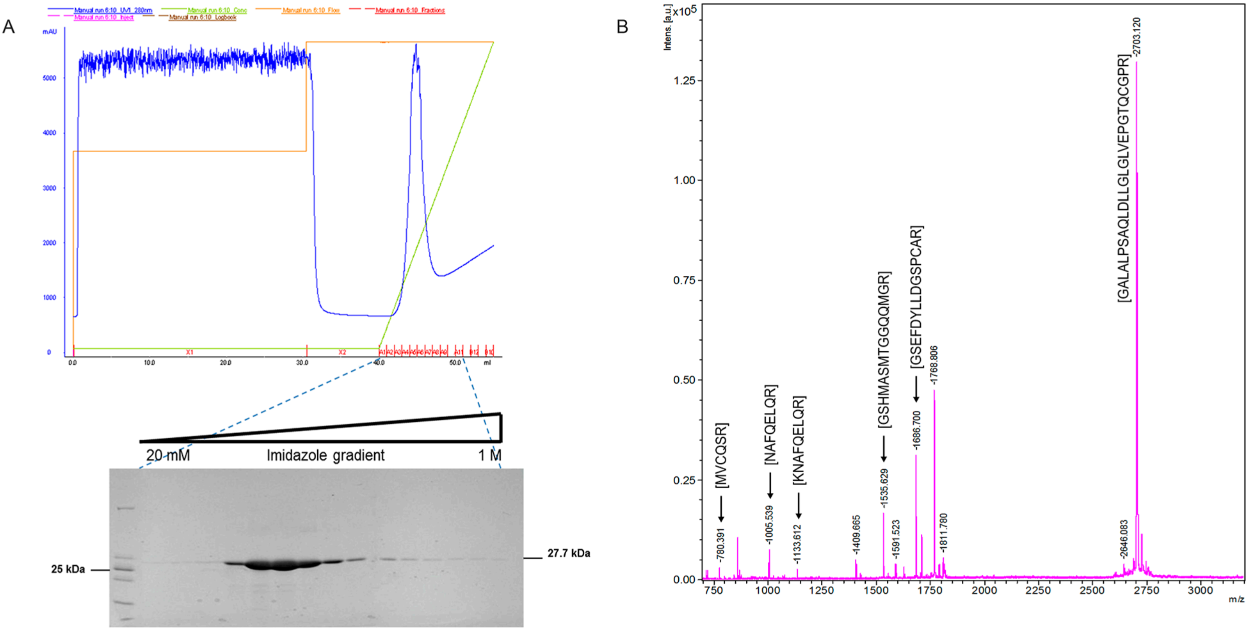

C

ADAM metallopeptidase domain 33, isoform CRA_d [Homo sapiens]

Query	Observed	Mr(expt)	Mr(calc)	ppm	Miss	Score	Expect	Rank	Unique	Peptide
1	2703.2661	2702.2588	2702.4425	-67.98	0	38	0.078	1	U	R.GALALPSAQLDLLGLGLVEPGTQCGPR.M

ADAM33-Rec sequence:
YLLDGSPCARGSGYCWDGACPTLEQQCQQLWGPGSHPAPEACFQVVNSAGDAHGNCGQDSEGHFLPCAGRDAL
CGKLQCQGGKPSLLAPHMVPVDSTVHLDGQEVTCRGALALPSAQLDLLGLGLVEPGTQCGPRMVCQSRRCRKNAF
QELQRCLTACHSHGVCNSNHNCHCAPGWAPPFCDKPGFGGSMDSGPVQAENHDTFL

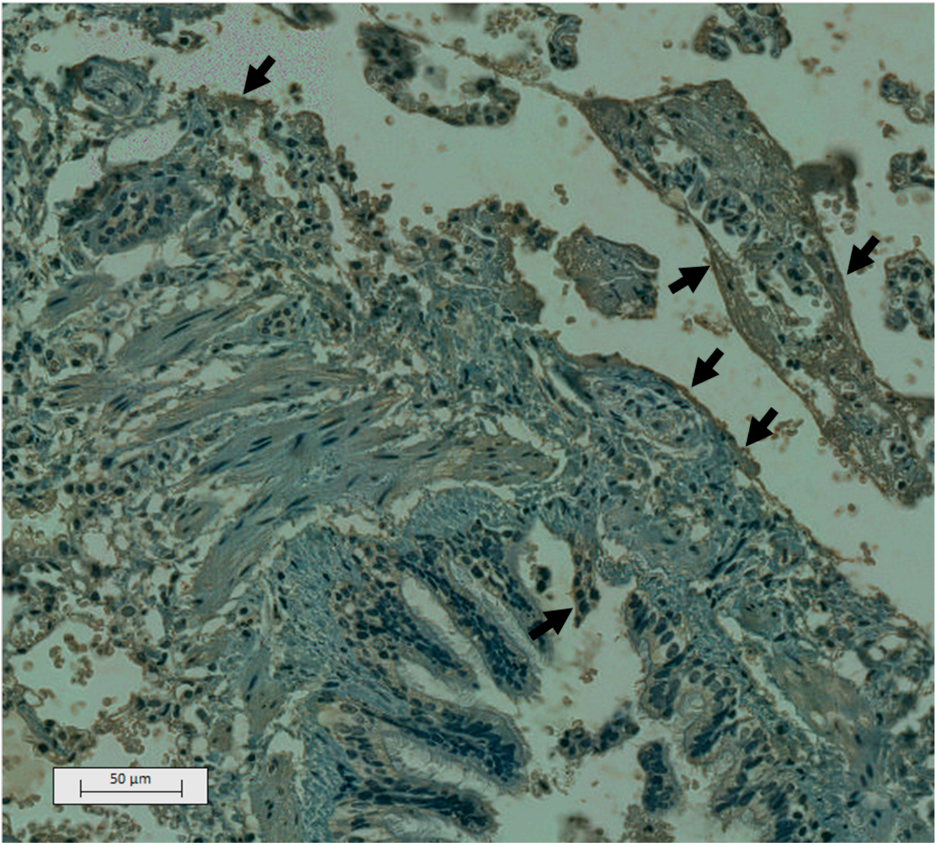
50 µm

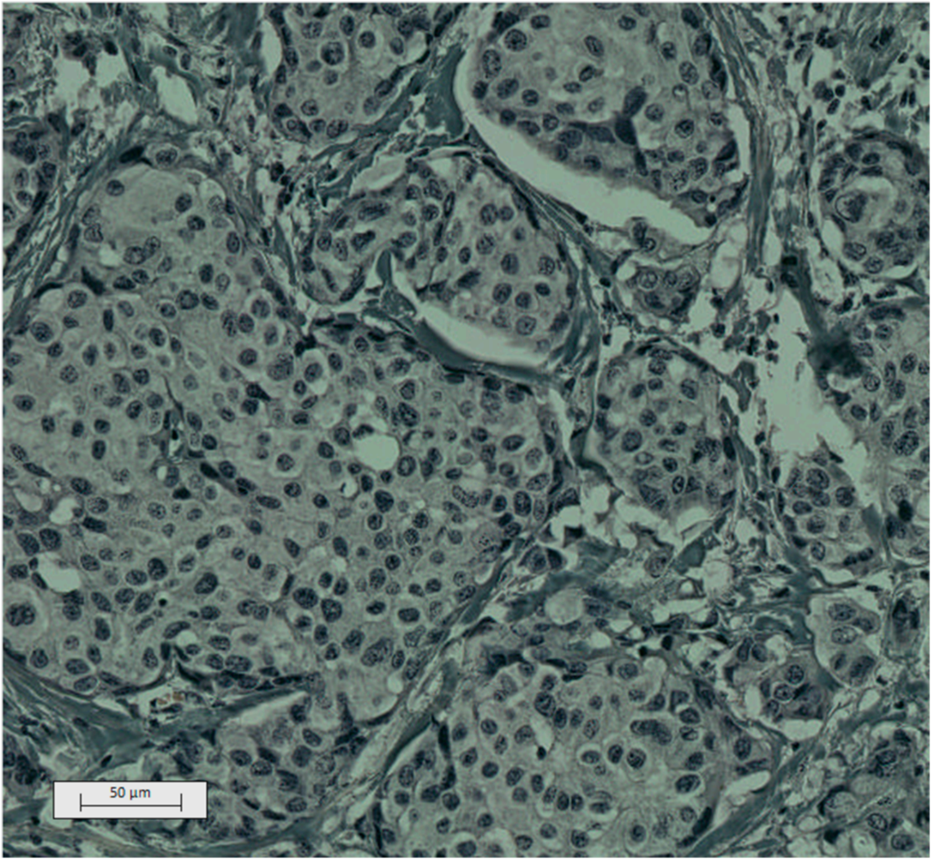
50 µm

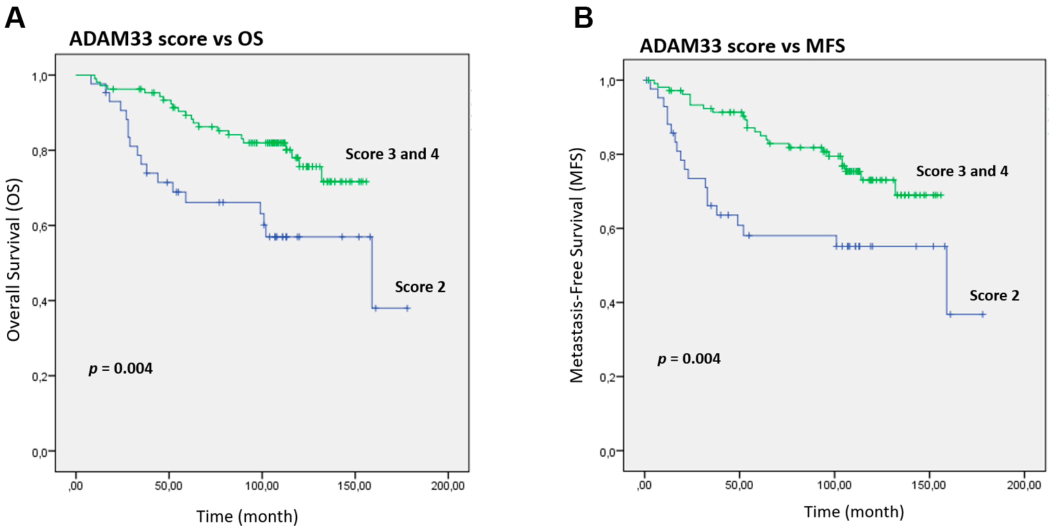
A
ADAM33 score vs OS
Overall Survival (OS)
1,0
0,8
0,6
0,4
0,2
0,0
,00
50,00
100,00
150,00
200,00
Time (month)
Score 3 and 4
Score 2
p = 0.004
B
ADAM33 score vs MFS
Metastasis-Free Survival (MFS)
1,0
0,8
0,6
0,4
0,2
0,0
,00
50,00
100,00
150,00
200,00
Time (month)
Score 3 and 4
Score 2
p = 0.004
